# Emerging Roles for Mammalian Target of Rapamycin (mTOR) Complexes in Bladder Cancer Progression and Therapy

**DOI:** 10.3390/cancers14061555

**Published:** 2022-03-18

**Authors:** Jianya Huan, Petros Grivas, Jasmine Birch, Donna E. Hansel

**Affiliations:** 1 Department of Pathology & Laboratory Medicine, Oregon Health & Science University, Portland, OR 97239, USA; huanj@ohsu.edu (J.H.); jasmine.birch@wsu.edu (J.B.); 2 Division of Medical Oncology, Department of Medicine, University of Washington School of Medicine, Fred Hutchinson Cancer Research Center, Seattle Cancer Care Alliance, Seattle, WA 98195, USA; pgrivas@uw.edu

**Keywords:** bladder cancer, urothelial carcinoma, mTOR, invasion, progression, inhibitor, targeted therapy

## Abstract

**Simple Summary:**

The mammalian target of rapamycin (mTOR) pathway promotes cell growth and metabolism in response to growth factors, nutrients, and cellular energy status. Dysregulation and mutation of the mTOR pathway can contribute to tumor initiation and progression. mTOR pathway abnormalities have been found in approximately 70% of urothelial carcinomas (UCs). This review summarizes recent advances in knowledge of the mTOR pathway, highlights the role of the mTOR pathway in UC, and explores the potential of the mTOR pathway as a therapeutic target in UC. This review also explores current preclinical studies and clinical trials targeting the mTOR pathway, and concludes with future directions of research that could more robustly define and target aberrant mTOR activity in bladder cancer.

**Abstract:**

The mammalian target of rapamycin (mTOR) pathway regulates important cellular functions. Aberrant activation of this pathway, either through upstream activation by growth factors, loss of inhibitory controls, or molecular alterations, can enhance cancer growth and progression. Bladder cancer shows high levels of mTOR activity in approximately 70% of urothelial carcinomas, suggesting a key role for this pathway in this cancer. mTOR signaling initiates through upstream activation of phosphatidylinositol 3 kinase (PI3K) and protein kinase B (AKT) and results in activation of either mTOR complex 1 (mTORC1) or mTOR complex 2 (mTORC2). While these complexes share several key protein components, unique differences in their complex composition dramatically alter the function and downstream cellular targets of mTOR activity. While significant work has gone into analysis of molecular alterations of the mTOR pathway in bladder cancer, this has not yielded significant benefit in mTOR-targeted therapy approaches in urothelial carcinoma to date. New discoveries regarding signaling convergence onto mTOR complexes in bladder cancer could yield unique insights the biology and targeting of this aggressive disease. In this review, we highlight the functional significance of mTOR signaling in urothelial carcinoma and its potential impact on future therapy implications.

## 1. Introduction

Bladder cancer constitutes the fourth most common cancer in men, with more than 83,000 new cases reported in the United States alone in 2021 [1]. Urothelial carcinoma (UC) accounts for more than 90% of all bladder cancer diagnoses [2] and encompasses a spectrum of disease findings, such as grade, stage, variant histology formation, and location within the urinary tract. Major categorical distinctions in UC include low- versus high-grade disease, and non-muscle-invasive versus muscle-invasive disease in the bladder. The greatest risks occur in the setting of high-grade disease, which is prone to progressive invasion, and in muscle-invasive bladder cancer, which harbors risk of metastasis in approximately 50% of patients [3,4]. For decades and until several years ago, the field of bladder cancer had remained stagnant, with Bacillus Calmette–Guérin (BCG) therapy and radical cystectomy (bladder removal) representing the main treatment options for high-grade, non-muscle-invasive bladder cancer and muscle-invasive bladder cancer, respectively. Fortunately, new research and clinical trial initiatives have dramatically expanded the options for patients, especially those with high-grade disease. The broader use of cisplatin-based chemotherapy, tri-modality therapy, immunotherapy, antibody drug conjugates, and targeted therapy approaches have improved therapeutic approaches for all patients [5,6]. However, gaps in our deeper understanding of bladder cancer biology and behavior remain.

One of the most robustly activated signaling pathways in UC is the mammalian target of rapamycin (mTOR) pathway [7,8,9]. While studies had been undertaken to analyze the impact of PTEN (phosphatase and tensin homolog) alterations in bladder cancer, the broad-scale analysis in bladder cancer afforded by the Cancer Genome Atlas Project highlighted a broader role for signaling through the mTOR pathway, also in advanced disease [10,11]. Activation of this pathway in bladder cancer occurs via either mutational change within the signaling pathway or upstream signaling activity through overexpressed or aberrantly expressed growth factors. Despite the increased activity of the mTOR pathway in bladder cancer, therapy based on mutational status with inhibitors of the mTOR signaling pathway have not necessarily proven successful in this disease. In this review, we first describe the mTOR signaling cascade, emphasizing differences between the mTORC1 and mTORC2 signaling arms, highlight cell signaling and genomic alterations, describe therapeutic strategies employed to target aberrant mTOR signaling, and conclude with future directions of research that could more robustly define and target aberrant mTOR activity in bladder cancer.

## 2. Bladder Cancer Overview

Bladder cancer represents the 10th most common cancer globally [12] and the 4th most common cancer in men in the United States [1]. Bladder cancer disproportionately affects men, with a male:female ratio of approximately 3:1 [1]. While individuals over the age of 55 are at greatest risk for bladder cancer, the disease can occur less commonly in young and middle-aged adults [13]. Smoking is a major risk factor for bladder cancer and has been identified in association with almost half of all cases of bladder cancer in the United States [14]. Environmental and occupational exposure to chemicals, such as arsenic, nitrosamine, aromatic amines, organosulfur compounds, polycyclic aromatic hydrocarbons, and other chemicals, also constitute known risk factors for bladder cancer [15,16]. Moreover, genetic predisposition (germline mutations, family history), chronic bladder inflammation/infections, frequent mechanical injury from catheterization [17], congenital anomalies, neurogenic bladder, diverticulum formation [18], and potentially reduced water intake [19] have all been demonstrated to increase the risk of this disease.

**Figure 1:**
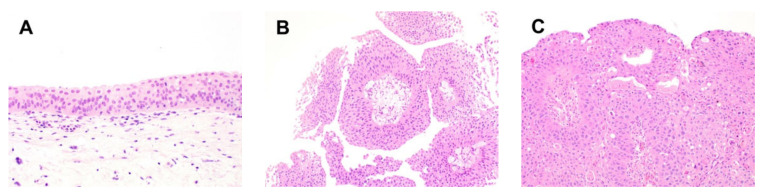
**Histology of urothelium.** (**A**) Normal urothelium is a polarized epithelial lining containing urothelial cells and surface umbrella cells; the urothelial lining is polarized, with a relatively uniform nuclear size, open chromatin, and limited to rare mitotic figures (200×). (**B**) Low-grade papillary urothelial carcinoma is a papillary neoplasm with thin fibrovascular cores lined by relatively polarized urothelium containing occasional hyperchromatic nuclei (100×). (**C**) High-grade papillary urothelial carcinoma, another papillary neoplasm, shows cellular disorganization and enlarged hyperchromatic nuclei with nuclear pleomorphism and abnormal localization of mitotic figures (100×).

Bladder cancer arises from alterations in the urothelium, the epithelial lining of the bladder. While cancerous alterations in the urothelium give rise to UC in over 90% of cases, other forms of bladder cancer can occur, including, but not limited to, squamous cell carcinoma, adenocarcinoma, and small cell carcinoma [2]. Urothelial carcinoma is diagnosed by light microscopy, with an emphasis on the grade and stage, each of which drives cancer behavior and therapy. To date, light microscopy remains the primary method to diagnose and segregate UC. Non-invasive tumors account for 80% of UC and consist of either low-grade or high-grade papillary UC or urothelial carcinoma in situ [20] (Figure 1). Low-grade papillary carcinoma is an exophytic lesion, containing thin fibrovascular cores. The urothelial lining retains polarity but shows scattered hyperchromatic nuclei caused by increased DNA content, and mild nuclear irregularities. Mitotic figures are limited to absent and, if present, are typically situated towards the basal layer. By contrast, high-grade lesions show more overt neoplastic changes at the architectural and cellular level, including disorganization, loss of polarity, loss of cellular cohesion, nuclear hyperchromasia, marked nuclear contour abnormalities, and mitotic figures aberrantly located in the upper layers of the urothelium. While high-grade papillary urothelial carcinoma demonstrates an exophytic appearance with thin fibrovascular cores, urothelial carcinoma in situ is a flat version of high-grade in situ neoplasia. Low-grade papillary urothelial carcinomas frequently recur but rarely evolve into high-grade lesions.

The major distinction in the management of high-grade UC is the depth of invasion into the bladder wall, or the pathological stage. In general, invasion is not compatible with the diagnosis of low-grade papillary UC [21]. In the setting of high-grade lesions, a subset of these lesions will progress to invasion. In these cases, invasion progresses stepwise into the lamina propria, followed by into the muscularis propria (detrusor muscle), the perivesical fat, and finally adjacent organs.

Clinically, lesions are often initially detected and removed by cystoscopy and are treated based on the grade and stage determined by pathology analysis. Low-grade papillary UC generally includes systematic follow-up with removal of recurrent lesions and, on occasion, intravesical therapy in patients in whom cancer control is challenging. By contrast, high-grade non-invasive lesions show high rates of recurrence and progress to invasive disease in approximately 50–70% of cases and are thus treated more aggressively [22,23]. For high-grade UC that remains restricted to the surface urothelium, such as high-grade papillary urothelial carcinoma or urothelial carcinoma in situ, or invades into the lamina propria, patients are managed with intravesical Bacillus Calmette–Guérin (BCG). BCG is an attenuated form of *Mycobacterium bovis* that elicits a robust immune response against cancer cells. For patients who develop invasion into the muscularis propria (detrusor muscle; muscle-invasive bladder cancer), recommendations include radical cystectomy, ideally preceded by cisplatin-based neoadjuvant chemotherapy in fit patients. Bladder preservation with tri-modality therapy (maximum TURBT followed by concurrent chemoradiation) is a common strategy in well selected patients based on criteria. Newer agents, such as immune checkpoint inhibition, targeted therapies, and antibody drug conjugates, are being investigated as therapeutic options in clinical trials in localized bladder cancer. 

The understanding of molecular and signaling pathway aberrations involved in low- and high-grade bladder tumorigenesis continues to rapidly evolve. Historically, low-grade papillary UC was characterized by frequent mutations in fibroblast growth factor receptor 3 (*FGFR3*), whereas high-grade UC by mutations in the tumor suppressor genes, such as retinoblastoma susceptibility gene (*RB1*) and tumor protein P53 (*TP53*) [24,25,26,27]. Research over the past decade has discovered a much broader array of alterations, including *ERBB2*, *MDM2*, *CDKN2A*, *KDM6A*, and *ARID1A* gene alterations in high-grade UC, key driver signaling pathways not previously recognized in low-grade UC (such as *PIK3CA*, *STAG2*, and RTK/RAS/RAF pathway genes), and gene expression differences across UC grades, stages, histology variants, and primary tumor locations [5,11,28,29,30]. Many of these alterations appear to have direct or indirect effects on the mTOR signaling pathway, suggesting that improved knowledge of the function of mTOR signaling in UC could yield insights into alternative therapy approaches. 

## 3. mTOR Cell Signaling Pathway

**Figure 2:**
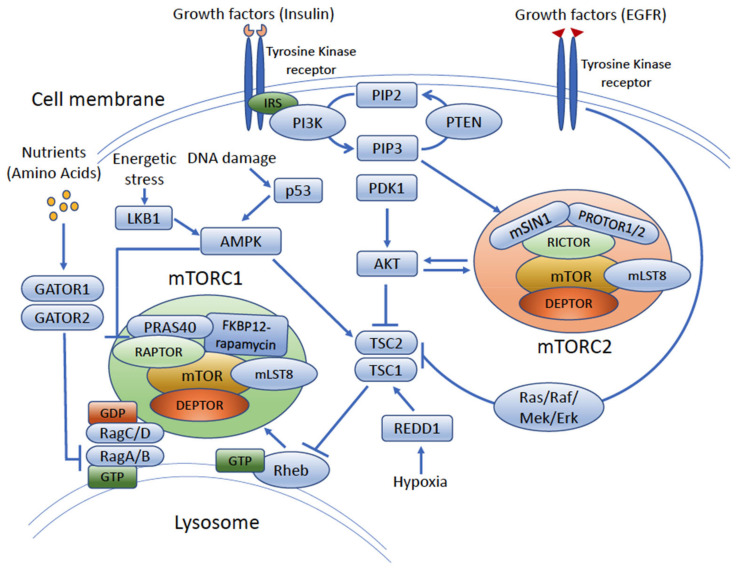
**The cellular signaling and regulation of the mTOR pathway**. Structurally, mTORC1 and mTORC2 share several protein components, including mTOR kinase, DEPTOR, and mLST8. Each complex also contains a couple of unique proteins. RAPTOR and PRSA40 are the components of mTORC1, where RICTOR and mSIN1 are the subunits of mTORC2. Both complexes integrate extracellular signaling through upstream PI3K/AKT and Ras/Raf/Mek/Erk signaling pathways to guide their own activation. mTORC1 is also regulated by the cellular status of amino acids, hypoxia, energetic stress, and DNA damage while mTORC2 mainly responds to growth factors. IRS, insulin receptor substrate; PI3K, phosphoinositide 3-kinases; PIP2, phosphatidylinositol (4,5)-bisphosphate; PIP3, phosphatidylinositol (3,4,5)-trisphosphate; PTEN, phosphatase and tensin homologue; PDK1, phosphoinositide-dependent protein kinase-1; AKT, protein kinase B; mTOR, mammalian target of rapamycin; RAPTOR, regulatory-associated protein of mTOR; DEPTOR, DEP-domain-containing mTOR-interacting protein; PRAS40, proline-rich AKT substrate 40 kDa; mLST8, mammalian lethal with SEC13 protein 8; RICTOR, rapamycin-insensitive companion of mammalian target of rapamycin; PROTOR1/2, protein associated with rictor 1 or 2; mSIN1, MAPK-interacting protein 1; TSC1/TSC2, tuberous sclerosis complex 1, 2; Rheb, Ras homolog enriched in brain; AMPK, AMP-activated protein kinase; GATOR1/2, GAP activity towards the Rags 1,2; LKB1, liver kinase B1; Ras, rat sarcoma kinase; RAF, rapidly accelerated fibrosarcoma kinase; ERK, extracellular signal-regulated kinases, Mek, MAPK/ERK kinase; EGFR, epidermal growth factor receptor; REDD1, regulated in development and DNA damage responses 1.

mTOR, which functions through either the mTORC1 or mTORC2 complex, may be activated by the upstream phosphoinositide 3-kinases (PI3K)/protein kinase B (AKT) signaling pathway, and by other factors. In AKT-driven signaling, the cascade begins at the cell membrane, where receptor tyrosine kinases bind growth factors, cytokines, and nutrients [7,31,32] (Figure 2). Activation of these cell surface receptors induces activation of PI3K. While there are three major classes of PI3K enzymes, only class I PI3K has been implicated in the tumorigenesis of bladder cancer [33]. Class I PI3K is a heterodimer kinase formed by a catalytic subunit with four genetic variants, including p110α (PIK3CA), p110β (PIK2CB), p110δ (PIK3CD), and p110γ (PIK3CG), and a regulatory subunit containing five isoforms: p85α, p55α, p50α, p85β, and p55γ [31,33]. Activated PI3K phosphorylates the membrane-anchored phosphatidylinositol-4,5-bisphosphate (PIP2) to phosphatidylinositol-3,4,5-trisphosphate (PIP3). The resultant PIP3 functions as a lipid messenger and subsequently leads to the activation PIP3-dependent protein kinase-1 (PDK1) and mTORC2, which results in the phosphorylation of Thr308 and Ser473 of AKT, respectively [34,35]. Phosphatase and tensin homolog (PTEN), a tumor suppressor critical in mTOR pathway signaling [36], can function to dephosphorylate PIP3 at this stage, essentially turning “off” the cell signaling cascade prior to activation of mTOR. PTEN loss has been shown in several studies to result in poor patient prognosis in bladder cancer and more aggressive UC behavior, suggesting it also functions as a tumor suppressor in this cancer type [37,38,39].

In cells in which PIP3 remains phosphorylated, it can activate downstream AKT, a key signaling factor and driver of cellular growth. AKT is a serine/threonine kinase that plays a critical role in normal cell physiology and tumorigenesis. AKT is activated by the phosphorylation of PDK1 at Thr308 and by mTORC2 at Ser473. AKT activation results in tuberous sclerosis protein 2 (TSC2) inactivation through phosphorylation of the GTPase-activating protein (GAP) domain on TSC2. This phosphorylation event results in disruption of the TSC complex, which consists of TSC1, TSC2, and TBC1D7, and consequent release of the inhibition on downstream mTOR activity [40,41] (Figure 2). Indeed, the TSC complex plays a unique role in the regulation of mTOR signaling and association of mTOR with the lysosome. Specifically, under static conditions, the TSC protein complex can negatively regulate the lysosomal GTP-bound Rheb (Ras homolog enriched in the brain) protein [41]. When TSC2 is phosphorylated by activated AKT, however, the TSC complex dissociates from the lysosomal surface and thereby relieves the inhibition of Rheb and results in the activation of mTORC1 [7,41]. Other upstream signaling factors can also phosphorylate TSC2 and activate mTORC1, including inflammatory cytokines and signaling through the Ras/Raf/Mek/Erk pathway [7,42,43,44]. 

## 4. mTOR Complexes: mTORC1 and mTORC2 

One of the major effects of PI3K/AKT signaling is activation of the mTOR signaling pathway, which typically exerts its effects via two major mTOR complexes called mTORC1 and mTORC2. The mTOR protein itself is a 289-kDa serine/threonine kinase in the PI3K-related kinase (PIKK) family that was discovered during studies on the immunosuppressive drug rapamycin [45,46]. mTOR functions in both normal and cancerous cells to regulate cell growth, metabolism, survival, and homeostasis by promoting anabolic processes, including protein, lipid, and nucleotide synthesis, and by suppressing catabolic processes, such as autophagy [7,32,47]. mTOR serves as a catalytic subunit in two distinct mTOR complexes: mTORC1 and mTORC2 [7,32]. mTORC1 and mTORC2 share several protein components, including mTOR, DEP domain-containing mTOR-interacting protein (DEPTOR), and mammalian lethal with SEC13 protein 8 (mLST8) [7,32] (Figure 2). DEPTOR is an endogenous inhibitor that regulates the activity of mTOR once mTOR is activated, thus resulting in a time-limited activation effect [7,32]. mLST8 is required for the stability and function of mTOR [7]. 

Despite these shared features, the unique proteins present in each complex drive distinct functions of mTORC1 and mTORC2. mTORC1 contains the scaffold protein regulatory-associated protein (RAPTOR). The association of RAPTOR with mTOR is essential for the phosphorylation of downstream target proteins, such as ribosomal protein S6 kinase 1 (S6K1) and eukaryotic translation initiation factor 4E-binding protein (4EBP1), both of which enhance protein translation. RAPTOR is also required for the lysosomal localization of mTORC1. The proline-rich AKT substrate of 40kDa (PRAS40) is a component and subtract of mTORC1 [48]. PRAS40 acts as an endogenous inhibitor to mTORC1 either by binding directly to mTOR kinase [49] or by association with RAPTOR, which results in the direct substrate competition of mTORC1 [48]. PRAS40 can be phosphorylated by AKT under insulin stimulation or mTOR kinase, which leads to the dissociation of PRAS40 from mTORC1 and relieves its inhibitory effect on the complex [48,50,51]. In the presence of rapamycin, a general inhibitor of mTOR, rapamycin binds to the 12 kDa FK506-binding protein (FKBP12), which then binds to the FKBP12–rapamycin-binding (FRB) domain of mTOR. As the result, the binding of FKBP12–rapamycin complex to the FRB domain of mTOR blocks the binding of mTOR to its substrate, which inhibits mTORC1 activity [52]. 

mTORC2 contains the unique proteins rapamycin-insensitive companion of mTOR (RICTOR), protein observed with RICTOR (Protor-1 and -2), and mitogen-activated protein kinase-associated protein 1 (mSIN1). Crystal structure analysis revealed that RICTOR blocks the binding of FKBP12–rapamycin complex to the FRB domain of mTOR, which results in insensitivity to rapamycin inhibition [53]. While Protor-1, Protor-2, and mSIN1 regulate mTORC2 functions through binding to RICTOR [42,43,54], one study suggested that mSIN1 has a phospholipid-binding pleckstrin homology (PH) domain, which can interact with PIP3 directly and assist mTORC2 assembly on the plasma membrane for phosphorylation of its physiological substrates [55]. 

## 5. mTORC1 Signaling Impacts UC Cell Size and Proliferation

**Figure 3:**
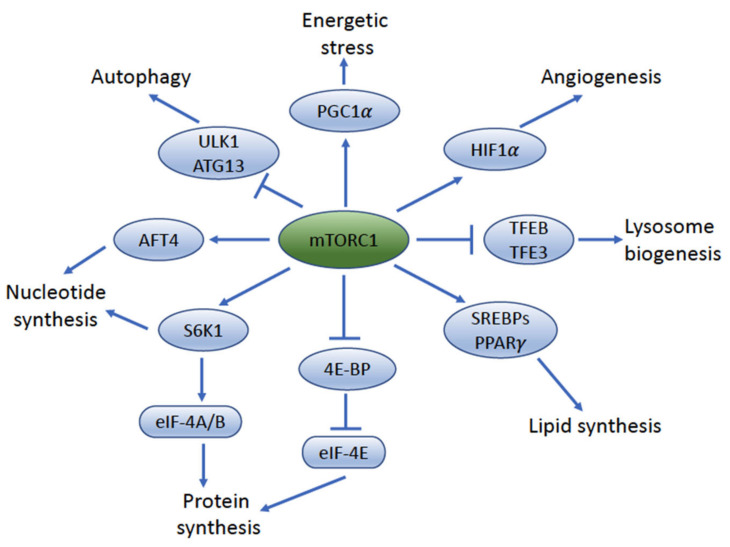
**The key downstream target molecules and function pathways of mTORC1**. mTORC1 is a master regulator of cellular anabolism and promotes cell metabolism, growth, and proliferation by regulating a variety of downstream effectors, including mRNA translation; biosynthesis of proteins, nucleotides, and lipids; autophagy; lysosomal biogenesis; and angiogenesis. 4E-BP, 4E-binding protein; eIF-4E, eukaryotic translation initiation factor 4E; S6K1, p70 S6 kinase 1; eIF-A/B, eukaryotic translation initiation factor 4A/B; HIF1α, hypoxia-inducible factor 1α; PGC1α, peroxisome proliferator-activated receptor γ coactivator 1-α; SREBP, sterol regulatory element-binding protein; PPARγ peroxisome proliferator-activated receptor-γ; TFEB, transcription factor EB; TFE3, transcription factor E3; ULK1, unc-51-like autophagy- activating kinase 1; ATG13, autophagy-related 13; AFT4, activating transcription factor 4.

mTORC1 is a master regulator of cellular anabolism and promotes cell metabolism, growth, and proliferation by regulating a variety of downstream effectors, including mRNA translation; biosynthesis of proteins, nucleotides, and lipids; autophagy; lysosomal biogenesis; and angiogenesis [7] (Figure 3). mTORC1 is activated when energy, growth factors, and macromolecular building blocks are sufficiently available [7]. As described above, activation of mTORC1 can be regulated by upstream growth factors through PI3K/AKT and Ras-MAPK signaling pathways. Nutrient status sensing occurs via the small GTPases Rags and Rheb, which help anchor mTORC1 at the lysosome in the presence of sufficient cellular amino acids, especially leucine and arginine [56,57]. This process is tightly regulated by the GATOR (GAP demonstrates activity towards the Rags) complexes GATOR1 and GATOR2. The GATOR complexes regulate the GDP/GTP states of the Rag-GTPase complexes RagA/B [58,59] (reviewed in [7,60]). It has been suggested that GATOR1 displays GAP activity toward RagA/B and functions as a negative regulator of mTORC1 signaling, whereas GATOR2 acts as an endogenous inhibitor to GATOR1 activity [7,60]. When amino acids are plentiful, GATOR2 is activated and inhibits GATOR1 activity. GATOR1 inhibition then allows RagA/B GTPase complexes to bind to RAPTOR and thereby recruit mTORC1 to the lysosomal surface, where mTORC1 can then be activated by GTP-bound Rheb [7] (Figure 2). 

Upregulation of mRNA translation and protein synthesis is a major function of mTORC1. Two important mTORC1 downstream target molecules involved in this process are the eukaryotic translation initiation factor 4E–binding protein 1 (4EBP1) and ribosomal protein S6 kinase 1 (S6K1). When mTORC1 is activated, it phosphorylates 4EBP1, interrupting its ability to form a complex with the eukaryotic translation initiation factor 4E (eIF4A) and thus allowing cell cycle progression and 5′ cap-dependent translocation of mRNAs [61,62]. mTORC1 also phosphorylates and activates S6K1 to stimulate translation of 5′ terminal oligopyrimidine tract mRNAs and promote initiation and elongation of translation [63,64]. The upregulation of mTORC1 by upstream oncogenic signaling allows cancer cells to meet the high demand needs by increasing both the cell size and cell number during tumorigenesis. 

In response to a positive oxygen and metabolic balance, mTORC1 can activate the sterol regulatory-element-binding proteins 1 and 2 (SREBP1/SREBP2), which control the expression of genes encoding proteins involved in lipid and cholesterol homeostasis [65]. Hypoxic conditions cause mTORC1 activity to result in phosphorylation of hypoxia-inducible factor 1 alpha (HIF-1α), thus switching the oxygen response within the cell [66]. 

mTORC1 is also a major negative regulator of autophagy, which is a critical intracellular degradation process to recycle damaged macromolecules and organelles under various conditions, including amino acid and energy deprivation [67]. Activation of mTORC1 by nutrients and growth factors leads to inhibition of autophagy through the direct phosphorylation and inhibition of UNC-5-like autophagy-activating kinase 1 (ULK1) [68] and autophagy-related gene 13 (ATG13) [69] within the ULK complex. mTORC1 can also phosphorylate ATG14L within the vacuolar protein sorting 34 (Vps34) complex, another complex that is crucial for autophagosome formation [70].

mTORC1 activity can be negatively regulated by multiple factors. In the setting of low cellular energy levels or a low ATP/high ADP balance, AMP-activated protein kinase (AMPK), a key sensor of cellular energy metabolism, can phosphorylate TSC2, and the latter subsequently inhibits Rheb activity, which results in the inhibition of mTORC1 [71]. AMPK can also phosphorylate RAPTOR, which blocks its ability to recruit and regulate the protein complexes, thus inhibiting mTORC1 [72]. mTORC1 can also be inhibited by low oxygen levels in the cytoplasm via activation of REDD1 (regulated in development and DNA damage response 1), BCL2 interacting protein 3 (BNIP3), and promyelocytic leukemia gene (PML). While REDD1 phosphorylates TSC2, activating it and leading to inhibition of mTORC1, BNIP3 and PML disrupt the Rheb–mTOR interaction, thus blocking activation [73,74,75]. Moreover, DNA damage can result in the activation of several p53 target genes, including AMPK-β1, PTEN, and TSC2, which inhibit mTORC1 activity [76]. 

In UC, mTORC1 sits at the center of a hub of dysregulated metabolic signaling. mTORC1 activity increases stepwise with the UC grade and stage [77] and causes increased UC cell size [78] and enhanced cancer cell proliferation [79]. In vitro studies using rapamycin, and siRNA targeting S6K1 and elF4E, in UC cell lines showed that treatment reduced cell viability and proliferation, suggesting mTORC1 regulates these cell processes in UC [80]. It has also been shown that higher mTOR activity, measured by 4EBP1 or S6K1 phosphorylation, correlates with a high risk of recurrence and poor survival in patients with UC [81]. In a mechanistic study of the mTORC1-S6K pathway, the findings showed that mTORC1-S6K pathway-phosphorylated RNF168, a E3 ubiquitin-protein ligase, inhibited its E3 ligase activity, and impaired its function in the DNA damage response, which led to an accumulation of unrepaired DNA and genome instability [82]. The study further suggested that loss of the tumor suppressor LKB1 induced hyperactivated mTORC1-S6K signaling, reduced RNF168 expression, and diminished the DNA damage response [82]. Moreover, another mechanistic study revealed that miR-29b, identified as a rapamycin-induced microRNA (miRNA), directly targeted tumor suppressor retinoid receptor beta (RARβ) to promote the tumorigenesis in *TSC2*-mutated mTORC1 hyperreactive cells as measured by cell proliferation, anchorage-independent cell growth, cell migration, and invasion [83]. This study further showed that miR-29b expression correlated with low RARβ expression in human cancer specimens using TCGA RNA-sequencing data from UC associated with *TSC* gene mutations [83]. This evidence supports that mTORC1 has an important role in UC development. Another study suggested that human telomerase reverse transcriptase (hTERT) is involved in immortalization and survival of cancer cells. The mutation of hTERT promoter occurred in 70% of bladder cancer patients associated with increased hTERT expression and poor prognosis [84]. In studying the mechanism of hTERT-derived tumorigenesis in UC, a recent study showed that the transcription factor TRIM28 (tripartite motif-containing 28) activated hTERT expression preferentially from the mutant promoter allele, and mTORC1-mediated phosphorylation of TRIM28 is required to activate hTERT transcription [85]. Ridaforolimus, a rapamycin analog, suppressed TRIM28 phosphorylation, hTERT expression, and UC cell viability [85].

## 6. mTORC2 Signaling Is a Key Driver of UC Migration and Invasion

**Figure 4:**
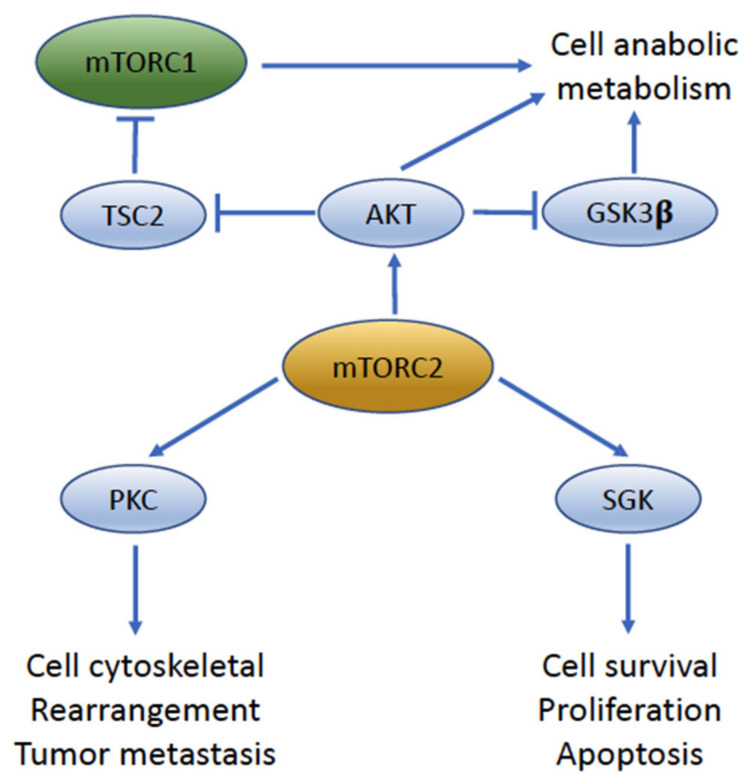
**The key downstream target molecules and function pathways of mTORC2.** mTORC2 activates the AGC (protein kinase A/protein kinase G/protein kinase C) family kinases PKC, AKT, and SGK to regulate the cytoskeleton, metabolism, and ion transport and promote cell survival. PKC, protein kinase C; SGK, serum- and glucocorticoid-induced protein kinase; GSK3β, glycogen synthase kinase 3β; TSC2, tuberous sclerosis complex 2.

mTORC2 has been less well-studied than the mTORC1 complex. The primary downstream effectors of mTORC2 are AKT [86], serum and glucocorticoid kinase 1 (SGK1) [87], and protein kinase C (PKC) [88], which are all members of the AGC (protein kinase A/protein kinase G/protein kinase C) protein kinase family (Figure 4). AKT is perhaps one of the best recognized targets of mTORC2 and the phosphorylation of Ser473 at AKT by mTORC2 can enhance AKT activity under physiological and pathological conditions [86,89]. mTORC2 can associate with ribosomes, where it can also phosphorylate nascent AKT at Thr405 to prevent co-translational AKT ubiquitination and stabilize the pool of AKT signaling [90]. mTORC2 can also phosphorylate PKCα and PKCζ to regulate actin cytoskeleton organization and thereby promote cytoskeletal organization and migration under normal physiological conditions, and cell invasion and metastasis under neoplastic conditions [91,92]. 

By contrast, the upstream regulation of mTORC2 is not fully understood, which may be partly may be due to its distribution in multiple subcellular compartments and its context-specific regulation [93,94,95]. Emerging evidence suggests that mTORC2 is regulated by growth factor signaling through the PI3K/AKT pathway and potentially regulated by the Ras signaling pathway via mSin1 [7,96]. In this scenario, growth factor activation of RTKs at the plasma membrane produces PIP3, which then interacts with the PH domain of PDK1 to activate PDK1. Activated PDK1 phosphorylates AKT at Thr308, resulting in AKT activation [97]. PIP3 can also bind directly to the PH domain of mSin1, which results in recruitment of mTORC2 and AKT to the plasma membrane where mTORC2 phosphorylates AKT at the Ser473 site [55,98]. In addition to growth factors, mTORC2 can also be directly activated by binding to ribosomes in melanoma and colon cancer cells [99]. Activation by some small Rho or Ras family GTPases, including Rac1, Rap1, and Ras, have also been suggested [100,101,102]. Intriguingly, Rho family GTPases are also reported to function as the downstream effectors of mTORC2 to promote actin cytoskeleton rearrangement and cancer cell invasion [103,104,105,106]. Our own study with a bladder cancer cell line (J82) showed that rictor gene silencing reduced Rac1-GTP levels to 60.2% of rictor-expressing cells, suggesting that Rac1 may be a major target of mTORC2 in bladder cancer [77]. 

Much of the additional work on mTORC2 activators and effectors in cancer has been specific to focused work in UC. In general, mTORC2 activity, as measured by the phosphorylation of AKT at Ser473, also shows a stepwise increase with UC grade and stage, with the highest levels of activity in invasive high-grade UC [77]. This finding is similar to the results seen in colorectal cancer, where both mTOR mRNA and protein levels were significantly increased in higher stage carcinomas [103]. The most important role mTORC2 appears to play in UC is to promote cancer cell migration and invasion; the understanding of the mechanism(s) related to these functions has been a primary focus of our laboratory over the past decade. 

In a recent study, we determined that mTORC2 activity was enriched within invadopodia, which are small invasive outgrowths of cancer cells. mTORC2 increased the expression of inducible nitric oxide synthase (iNOS), which co-localizes to invadopodia, and the local production of nitric oxide, which induced actin cytoskeleton rearrangement and additional invadopodia formation [107]. Thus, the nitric oxide produced via this mechanism suggests a feed-forward pro-invasive mechanism in UC. 

Given the limitations on knowledge of downstream mTORC2 signaling targets, we also recently undertook a mass spectrometry and reverse-phase protein array (RPPA)-based parallel phosphoproteomics study to discover new categories of downstream signaling factors of mTORC2 in UC. This study identified a wide range of cellular processes that were specifically regulated by mTORC2, including those involved in cell motility (such as autocrine motility factor receptor), cytoskeletal arrangement (such as adducin and cofilin), and cell and focal adhesion (such as FAK, SRC, N-cadherin, b-catenin, ezrin, talin-1, liprin α-1, liprin β-1, TJP1/ZO-1, and snail) [108]. Caveolin-1 (Cav-1), which can regulate caveolae formation through the. regulation of Cav-1 expression and/or Cav-1 Y14 phosphorylation, was a target of mTORC2. Functional studies suggested that mTORC2 could utilize Cav-1 regulation to secondarily impact the expression and/or stability of caveolar-associated receptor tyrosine kinases (RTKs) important for bladder cancer growth [108]. Analysis of 53 high-grade bladder carcinomas associated with metastases and also provisional TCGA RNA sequencing data suggests a strong association between high CAV1 expression and reduced survival [108]. Indeed, the ability of mTORC2 to regulate RTK expression, which are known to stimulate the mTORC1 response, indicates an intricate cross-talk between the mTORC1 and mTORC2 arms of the signaling cascade, possibly representing a balance between pro-metabolic mTORC1-driven proliferation pathways and pro-invasive mTORC2-driven process extension. 

Identification of upstream activators of mTORC2 in the setting of UC has also been somewhat limited. Recently, the urokinase receptor (uPAR) has been shown to be an upstream activator of mTORC2 in invasive bladder cancer and functions in a PTEN-dependent manner [109]. uPAR has also been shown to have an association with poor survival in UC patients [110]. uPAR is highly expressed in both non-invasive (54–71%) and invasive (94%) bladder cancers. uPAR knockdown in three highly invasive bladder cancer cell lines (T24, J82, and UM-UC-3) showed mTORC2 inhibition, reducing cell migration [109]. It has been suggested that uPAR could be used as a prognostic biomarker for non-invasive bladder cancer patients [111]. Another mTORC2-activating protein in UC is transforming growth factor-β (TGF-β). While TGF-β signaling can affect multiple signaling pathways within the cell, human bladder cancer cells showed an ability of TGF-β to induce phosphorylation of AKT at Ser473, increase bladder cancer cell migration, and impact epithelial-to-mesenchymal transition (EMT) during cancer progression [105,112]. Finally, cellular energy status may also affect mTORC2 activity. One recent study suggests that under acute energetic stress, mTORC2 can be activated directly by AMPK, which results in an increase in mTORC2 activity and cell survival. This event is independent of AMPK-mediated suppression of mTORC1 [113]. 

## 7. Genetic Alterations and Dysregulation of mTOR Signaling in Urothelial Carcinoma

In addition to cell signaling studies, mTOR pathway components in UC have also been analyzed through a variety of genomic approaches. The Cancer Genome Atlas (TCGA) project analyzed DNA, RNA, and protein data from 131 high-grade muscle-invasive bladder cancer specimens and identified significant genetic alterations in 32 genes, including mutations involving the PI3K/AKT/mTOR pathway (42% of cases) and receptor tyrosine kinase/mitogen-activated protein kinase (RTK/MAPK) pathway (44% of cases) [11]. Mutations in *PIK3CA*, which encodes the PI3K p110α subunit, are common in non-invasive bladder cancers (25–48% of cases) while mutations of *PIK3RI*, which encodes the p85α subunit, are less common [31,114,115,116]. Previous studies suggest that PIK3RI mutations are only present in approximately 1% of UC cases, whereas *PIK3CA* mutations are present in 17–25% of muscle-invasive bladder cancer cases [11,117]. 

The TCGA study showed that about 10% of muscle-invasive bladder cancer demonstrated overexpression of *AKT3* [11]. In the extended TCGA study that included 412 muscle-invasive bladder cancer samples, results from reverse-phase protein array (RPPA) proteomic data showed 5 distinct functional clusters associated with different protein expression profiles, pathway activities, and overall survival [28]. Among them, RPPA clusters 4 and 5 had high epithelial-mesenchymal transition (EMT) scores and relatively high levels of Cav-1 and RICTOR, a key component of mTORC2 [28]. Interestingly, our own study demonstrated a unique role for mTORC2-mediated regulation of caveolae formation through Cav-1 expression in actively migrating bladder cancer cells [108]. 

PTEN is an upstream negative regulator of mTOR signaling. PTEN mutations and loss of heterozygosity (LOH) are commonly seen in invasive urothelial cancers, where they have been associated with increased recurrence of bladder cancer [37,118]. Although *PTEN* mutations are only found in 3–4% of muscle-invasive bladder cancer cases [11,119], loss of PTEN protein expression is commonly observed in 39–94% of patients [38,114,120]. PTEN immunohistochemical expression appears to be decreased in bladder cancer specimens relative to normal urothelium, with one study showing loss of PTEN in up to 67% of bladder cancer specimens, as compared to 22% in in the adjacent non-neoplastic tissue [25]. 

Tuberous sclerosis proteins 1 (TSC1) and tuberous sclerosis proteins 2 (TSC2) form the TSC complex, which serves to reduce mTORC1 activity (Figure 2). The *TSC1* and *TSC2* genes are either inactivated by mutation, deletion, or both, such that they no longer allow the complex to regulate mTOR signaling [24]. A study of gene mutations in UC suggests that *TSC1* mutations occur in 12% of cases while *TSC2* mutations occur in only 3% of cases [117]. Other studies showed that LOH leading to decreased expression of TSC1 or TSC2 was present in 40–50% and 15% of muscle-invasive UC, respectively [114,121]. The inactivation of either of these proteins inhibits the formation of the TSC complex, which allows mTOR to continue functioning without regulation. 

Increasing evidence suggests that the high frequency of dysregulation of mTOR signaling could result from genetic alterations in upstream molecules, including various RTKs. Mutation of *FGFR3* is commonly associated with non-muscle-invasive bladder cancer (72–74%) and confers a favorable prognosis [122,123]. By contrast, *FGFR3* mutation is much less common in muscle-invasive bladder cancer, occurring in 15–20% of cases [108]; however, FGFR3 protein overexpression was demonstrated in up to 40% of these cases, suggesting an alternate mechanism to activate FGFR3 in this setting [124]. Epidermal growth factor receptor (EGFR), another receptor tyrosine kinase, shows overexpression in up to 74% of bladder cancer specimens, as compared to the relatively low expression level seen in normal urothelium [125,126,127]. Human epidermal growth factor receptor 2 (HER2) protein overexpression is reported in up to 45 % of bladder cancer specimens, with no expression found in normal urothelium [126,127]. In our own study with 53 invasive high-grade UCs and 42 paired lymph node metastases, the results showed that HER2 protein overexpression occurred in 19 UCs (36%) and 14 metastases (30%), with an 88% concordance rate between UCs and matched metastases. Moreover, amplification of *HER2* was present in only approximately 10% of cases, often associated with *MYC* amplification, suggesting more than one mechanism can increase HER2 protein expression [128]. Finally, insulin-like growth factor 1 receptor (IGF1R) overexpression was present in 62% of invasive UC [129]. In aggregate, these findings suggest that dysregulation of growth factor receptors may be critical in promoting the proliferation, differentiation, and survival of UC cells and may serve as potential therapeutic targets, especially FGFR3. 

Although the TCGA study showed that the genomic alteration of the PI3K/AKT/mTOR pathway occurred in 42% of invasive bladder cancer cases [11], there were few cancer-associated mutations that were directly linked to the *MTOR* gene itself. One study analyzed publicly available tumor genome sequence data from more than 400 samples, and identified 33 mutations in *MTOR* related to hyperactivation, which can strongly activate the downstream effectors (4EBP1 and S6K1) to promote the tumorigenesis [130]. Interestingly, in investigating how the major regulators (4EBP1 and S6K1) downstream of mTOR hyperactivation may specifically change gene expression profiling that promote tumorigenesis, a ribosomal profiling study suggested that the hyperactivation of mTORC1 specifically led to an increase in the expression of a group of genes, including Y-box-binding protein 1 (YB1), vimentin, metastasis-associated 1 (MTA1), and CD44, which are involved in cell invasion and metastasis [131]. Another study using the Catalogue of Somatic Mutations in Cancer (COSMIC) database (up to February 2019) revealed that the somatic mutation of *MTOR* was observed in 5.1% of urinary tract cancers (58/1138) whereas protein overexpression was 6.6% (27/408) [132]. Finally, in a gain-of-function mutation study, the authors mutated highly conserved amino acid residues within the critical domains of human mTOR, and then expressed the mutants in NIH3T3 cells. Subsequent in vitro studies showed that the mutations led to a strong activation of mTOR/S6K1 signaling, induced cell transformation, and enhanced invasion. After subcutaneous inoculation of the transfected cells in athymic nude mice, the results showed that the mutants caused rapid tumor formation and growth. These data suggested that mutated mTOR alone may directly lead to cancer pathogenesis although similar studies remain to be performed in UC cells [133]. 

## 8. Potential Targets of the PI3K/AKT/mTOR Pathway in Urothelial Cancer

Due to the frequency of alterations in the PI3K/AKT/mTOR signaling pathway, its role in promoting cancer cell growth and invasion, and integrative analysis data, targeting of this signaling cascade has a robust rationale with wide-ranging therapeutic implications [134]. Recent studies suggest that mTOR and phosphorylated mTOR expression in bladder tumors is correlated with an aggressive pathology [43,135,136]. However, early studies targeting this pathway in bladder cancer were disappointing, with responses seen only in a very small subset of patients. This raises the possibility that downstream effectors of mTOR or new upstream activators could serve as better alternative targets. Alternatively, more detailed in-depth characterization of individual cancers may provide a pathway to more rational and “customized” selection of therapeutics, similar to that shown in cell line models [137]. Finally, approaches that target the factors that act via PI3K/AKT/mTOR-dependent mechanisms and may synergize with Ras signaling (such as HRAS and KRAS), and FGFR3 signaling pathways, may provide broader options. Furthermore, the opportunity to broaden the gamut of mTOR signaling in the context of immunotherapeutic regimens may hold additional possibilities, although this is beyond the scope of the current article. 

### 8.1. PI3K Inhibitors

Given the critical role of PI3K in cellular transformation and the development of cancer, various class I PI3K isoform-specific inhibitors have been developed and studied in clinical trials [138,139]. Most PI3K inhibitors are pan-PI3K or PI3K isoform-specific ATP-competitive inhibitors (Table 1). Some of these inhibitors, including copanlisib (BAY 80-6946) (a pan-class I PI3K), alpelisib (BYL-719) (a PI3Kα-specific inhibitor), buparlisib (BKM-120), and pilaralisib (GDC-0941), have been studied in several trials and show modest efficacy and substantial toxicity [139,140,141]. However, most of these inhibitors have been primarily studied in clinical trials of leukemia and breast cancer, with far fewer studies in UC. 

Preclinical data using an in vitro model suggested that pictilisib can significantly reduce the tumor cell viability by inducing G1 cell arrest and apoptosis in multiple UC cell lines harboring either the PIK3CA mutation alone or with a co-existing NRAS mutation [142]. A recent animal study showed that buparlisib could significantly inhibit tumor growth and promote infiltration of human CD45+ cells and CD3+ T cells in humanized mice xenografted with PIK3CA-mutated human bladder cancer cells [143]. Data from clinical trials is more limited. A phase I study with pictilisib in advanced solid tumor, including advanced UC, showed that the drug had a favorable safety profile and preliminary antitumor activity [144]. In the recent phase II clinical trial, buparlisib was studied in patients with platinum-resistant metastatic UC (NCT01551030), showing modest clinical activity in patients whose tumors harbored TSC1 loss of function alterations and substantial toxicity of the drug [145]. 

### 8.2. AKT Inhibitors

AKT acts as a major signaling node within the PI3K/AKT/mTOR pathway and has a dominant effect on the activation of mTORC1 signaling. Several ATP-competitive pan-AKT inhibitors have been developed and evaluated in various tumor types, including UC [146,147]. Of special interest are the allosteric pan-AKT inhibitors, such as MK-2206 and AZ7328, which showed potent and selective AKT inhibition and lower toxicity in preclinical studies with human BC cell lines [148,149]. One study showed that treatment with pirarubicin (THP), a chemotherapeutic drug, can significantly increase the phosphorylation of both AKT and Erk1/2 in both murine and human BC cell lines. MK-2206 alone or in combination with AZD6244 (Erk1/2 inhibitor) can remarkably sensitize bladder cancer cells to THP treatment [150]. Borussertib, another allosteric pan-AKT inhibitor, was tested in vitro using eight different cancer cell lines, including KU-19-19 bladder cells harboring aberrations in the PI3K/AKT and RAS/MAPK pathways. Borussertib selectively inhibited the proliferation of PI3K/PTEN-mutated cell lines and downregulated AKT-mediated signaling [151]. Another recent study showed that capivasertib (AZD5363) inhibited cell proliferation in several human BC cell lines [152].

The results from the early phase clinical trials demonstrated an overall manageable safety profile and preliminary efficacy in various tumors [153,154,155]. In recent years, the first generation of ATP-competitive pan-AKT inhibitor, ipatasertib (GDC-0068), was evaluated in phase Ib clinical trials with various solid tumors, including advanced or metastatic UC. The results showed that ipatasertib in combination with chemotherapy or hormonal therapy was well tolerated, with a safety profile consistent with that of ATP-competitive AKT inhibitors [156]. There are unmet needs for clinical study in evaluating the efficacy of AKT inhibitors in UC.

**Table 1:** Selected inhibitors evaluated in the preclinical studies and clinical trials related to UC.

Inhibitor	Target	Disease Status	Study/Trial	Reference
Copanlisib	Pan-PI3K	Advanced UC	Phase I	[157]
Alpelisib	PI3Kα	Advanced UC	Phase Ib	[158]
Buparlisib	Pan-PI3K	Metastatic UC	Phase II	[145]
Pilaralisib	Pan-PI3K	Advanced UC	Phase I	[144]
MK-2206	Allosteric pan-AKT	UC cell lines	Preclincial	[148]
AZ7328	Allosteric pan-AKT	UC cell lines	Preclincial	[149]
Borussertib	Allosteric pan-AKT	UC cell lines	Preclincial	[151]
Capivasertib	ATP-competitive pan-AKT	UC cell lines	Preclincial	[152]
Ipatasertib	ATP-competitive pan-AKT	Advanced/metastatic UC	Phase Ib	[156]
Everolimus	Allosteric mTOR	Advanced/metastatic UC	Phase II	[159,160,161,162]
Temsirolimus	Allosteric mTOR	Metastatic UC	Phase II	[163,164]
ABI-009	Allosteric mTOR	BCG refractory NIMBC	Phase I/II	[165]
OSI-027	mTORC1, mTORC2	UC cell lines	Preclinical	[166]
Dactolisib	Dual PI3K/mTOR	UC cell lines	Preclinical	[167]
Sapanisertib	mTORC1, mTORC2	Metastatic UC	Phase II	[168]
Vistusertib	mTORC1, mTORC2	UC cell lines Advanced UC	Preclinical Phase Ib	[152] [169]
Erdafitinib	FGFR1–4	Metastatic UC	Phase II	[170]
AZD4547	FGFR1–3	Advanced UC	Phase Ib	[169]
Infigratinib	FGFR1–3	Advanced UC	Phase I	[171]
Pemigatinib	FGFR1–3	Metastatic UC	Phase II	[172]
Rogaratinib	FGFR1–4	Metastatic UC	Phase II/III	[173]

### 8.3. mTOR Inhibitors

mTOR pathway inhibitors are not new cancer therapeutic agents given the importance of mTOR signaling in cell growth, survival, and proliferation. Rapamycin (sirolimus) is an allosteric inhibitor of mTOR, and is now used for cancer treatment, anti-aging efforts, and other clinical uses [174,175,176,177]. Three other FDA-approved analogs of rapamycin (rapalogs) are being used as well: everolimus, temsirolimus, and ridaforolimus [178]. These rapalogs are generally considered the first generation of mTOR inhibitors. Everolimus inhibits bladder cell line proliferation and promotes antitumor activity in urothelial cell lines, suggesting that it could be active against this cancer type [178]. A number of phase I/II studies were conducted to evaluate the feasibility and toxicity of everolimus in urothelial carcinoma in situ and in advanced UC patients; however, these trials reported substantial side effects [179,180,181]. In two additional phase II studies, the results showed the clinical benefit of everolimus in a subset of patients with advanced or metastatic UC [159,161]. In a phase II trial, 45 patients with advanced UC were enrolled and nearly half of the patients (48.9%) responded to temsirolimus treatment; however, 94% of the patients experienced some degree of drug toxicity and nearly 53% of patients had a severe adverse event [164]. Interestingly, a relevant study showed that patients with advanced bladder cancer harboring *TSC1* somatic mutations might have a higher chance of having a response with everolimus, but further validation is needed. This suggests that genomic alterations in UC may potentially provide useful information for mTORC1-directed therapies [182]. Data from these early trials, however, highlight the potential risk of high-dose mTOR inhibition, which may cause toxicity and metabolic disturbances, such as insulin resistance, drug-induced pneumonitis, hypophosphatemia, and hyperlipidemia [183,184].

Second-generation mTOR inhibitors are small molecular ATP analogues, including ATP-competitive mTOR inhibitors (Onatasertib and OSI-027) and PI3K/mTOR dual inhibitors (INK-128 and NVP-BEZ235). These inhibitors can directly bind or act on the ATP-binding sites of mTOR or PI3K to produce competitive inhibitory effects [185]. ATP-competitive mTOR inhibitors are highly selective to mTOR, which can inhibit both mTORC1 and mTORC2. The advantage of these types of mTOR inhibitors is that they inhibit mTORC1 better due to decreased AKT phosphorylation by mTORC2. In one preclinical study, three invasive bladder cancer cell lines (T24, HT1376, and UM-UC-3) were treated with OSI-027, which inhibited the phosphorylation of both mTORC1 and mTORC2 pathway components (pAKT, p4EBP1, and pS6K1) and reduced cell proliferation. OSI-027 also exhibited antitumor synergy when in combination with lapatinib in UC in vitro [166]. In a recent phase II clinical trial (NCT03047213), Sapanisertib, a potent inhibitor of mTORC1 and mTORC2, failed to show clinical benefits in a small group of patients (n = 13) with *TSC1*- or *TSC2*-mutated metastatic UC. This result led to the suggestion that future studies with mTOR inhibitor or other targeted agents in the mTOR signaling pathway should investigate molecular alterations beyond *TSC1* or *TSC2* [168].

Because the class I PI3K catalytic subunit (p110) shares high structural similarity with mTOR, a strategy to develop an inhibitor with dual inhibitory functions has gained momentum. In preclinical and early clinical studies, a number of dual inhibitor candidates have shown promise [175]. NVP-BEZ235 (Dactolisib) is an oral mTOR/PI3K dual inhibitor [186] and has been shown to suppress tumor cell growth by inducing cell cycle arrest and caspase-dependent apoptosis in eight different human bladder cancer cell lines [167]. A strong synergistic effect was reported when NVP-BEZ235 was used in combination with cisplatin in cisplatin-resistant human bladder cancer cell lines [167]. Another recent study using seven human bladder cancer cell lines in a 3D high-throughput screening platform showed that the combination of AKT inhibitor (AZD5363) with a dual PI3K/mTOR inhibitor (NVP-BEZ235) or an mTOR inhibitor (AZD2014) exhibited synergistic effects on cell viability and colony formation [152]. 

Other than targeting the PI3K/AKT/mTOR pathway, a recent study suggested that the inhibition of S6K1 and 4EBP1, its downstream effector proteins, helps control bladder tumor growth and progression [187]. These therapies suggest that while inhibitors targeting the PI3K/AKT/mTOR pathways and its downstream proteins can produce antitumor effects, they may possibly be enhanced by combining them with various other cancer treatments. Ongoing efforts to expand our mechanistic knowledge of mTOR signaling in the context of UC may provide additional opportunities for synergistic targeting of the PI3K/AKT/mTOR pathway in this disease. 

### 8.4. FGFR Inhibitors

In recent years, FGFR inhibitors have emerged as a promising treatment for patients with platinum-refractory metastatic UC [188,189]. Given that FGFR signaling can impact downstream mTORC1 and mTORC2 activity, evaluation of FGFR inhibitors in the context of mTOR blockade is relevant to pathway targeting. 

Activated FGFRs can signal through the PI3K/AKT/mTOR and RAS/RAF/MEK/ERK pathways, and thereby stimulate cell growth, differentiation, survival, and angiogenesis [188,190,191,192]. It has been suggested that UC has the strongest association to FGFR gene alterations when compared to all other cancers [193]. In 2019, FDA granted accelerated approval to the FGFR inhibitor Erdafitinib for patients with locally advanced or metastatic UC that harbored susceptible *FGFR3* or *FGFR2* genomic alterations (activating mutation or fusion/rearrangement) and who had progression on platinum-based chemotherapy. This approval was based on a single-arm phase II trial of 99 patients with platinum-refractory metastatic UC, which showed that Erdafitinib yielded an objective response rate (ORR) of 40% (95% CI 31–50), including 3% with a complete response and 37% with a partial response. The ORR was 49% among 74 patients with FGFR mutations, 16% in the 25 patients with FGFR fusions, and 59% in the 22 patients who previously failed in anti-PD-1/PD-L1 immunotherapy [170]. The study showed that at a median follow-up of 11.2 months (IQR 8.2–15.6), median progression-free survival was 5.5 months (95% CI 4.2–6.0) and, at a median follow-up of 11.0 months (IQR 8.5–14.1), median overall survival was 13.8 months (95% CI 9.8–not reached) [170]. The study also reported that 46% of patients had treatment-related adverse events of grade 3 or worse. The most frequently reported adverse events (grade ≥3) were hyponatremia (11%) and stomatitis (10%). No treatment-related deaths were reported [170]. In a long-term follow-up phase II trial, the efficacy and safety of erdafitinib were evaluated in 101 patients who had locally advanced or metastatic urothelial carcinoma with FGFR3 gene mutations (R248C, S249C, G370C, Y373C) or FGFR gene fusions (FGFR3-TACC3, FGFR3-BAIAP2L1, FGFR2-BICC1, FGFR2-CASP7) [194]. The study showed that at a median follow-up of 24 months (IQR 22.7–26.6), the ORR with erdafitinib was 40% (95% CI 30–49), including a complete response rate of 4.0% and a partial response rate of 36.0%. Median progression-free survival was 5.5 months (95% CI 4.3–6.0), and the 12-month progression-free survival rate was 21% (95% CI 13–29) [194]. There were no new safety concerns reported in the follow-up study compared with the safety data from the primary trial [194]. An ongoing phase III trial with an estimated accrual target of 631 patients (NCT03390504) is evaluating the efficacy of erdafitinib versus chemotherapy agent (docetaxel or vinflunine) or pembrolizumab (anti-PD-L1) in patients with advanced UC harboring selected *FGFR* genomic aberrations who had progression on/after prior therapy [188]. Moreover, another clinical trial aimed to evaluate the safety and efficacy of durvalumab (anti-PD-L1) in combination with targeted therapies, including AZD4547 (pan-FGFR inhibitor) or olaparib or vistusertib (mTORC1/mTORC2) inhibitor), in advanced platinum-refractory UC but did not meet the predefined threshold for adequate efficacy to move any of the tested combinations to a larger phase III trial [169]. 

Studies showed that UC harboring FGFR3 S249C-activating mutation are not always sensitive to FGFR inhibitors because of their possible dependence also on EGFR signaling rather than only on FGFR3 signaling, which is repressed. Furthermore, UC cells harboring FGFR3-TACC3 fusions acquire resistance to FGFR inhibitors through the upregulation of EGFR/HER3-dependent PI3K-AKT signaling [195,196]. This may suggest that targeting both PI3K/AKT/mTOR- and FGFR-driven pathways could offer a potentially synergistic effect in combating drug resistance in UC treatment. Indeed, a recent study in ovarian cancer suggested that a combination of the FGFR inhibitor infigratinib and the mTOR inhibitor rapamycin induced remarkable cell cycle arrest and apoptosis in ovarian cancer cells, and reduced tumor size in the xenograft animals after the treatment. Such an experimental effect was not observed in the infigratinib or rapamycin alone group [197]. Moreover, in a separate clinical trial on 112 patients with metastatic breast cancer who were treated with a non-selective FGFR inhibitor and the mTOR inhibitor rapamycin, higher response rates were achieved in patients with simultaneous amplification in FGFR/FGF signaling and alterations in the PI3K/AKT/mTOR pathway who were treated with both of inhibitors [198]. Such combinatorial approaches may be tested across cancer types in the NCI-Combo-Match trial, which is under development; this type of additive and/or synergistic approach may be valuable ultimately in the context of UC. 

## 9. Future of Targeting mTOR in Bladder Cancer Therapy

While the progress of mTOR inhibition is ever changing and advancing, there is still more work to do. Overcoming resistance to mTOR inhibitors and using metabolic derangements of cancer cells for active targeting of the mTOR pathway may be of value [199,200]. A recent study that analyzed the mechanisms of resistance in temsirolimus-resistant and -sensitive (parental) RT112 and UMUC3 bladder cancer cell lines showed that the drug resistance was associated with increased tumor growth and invasion, enhanced expression of cell cycle proteins, and elevated phosphorylation of AKT/mTOR signaling proteins. The data also showed that the levels of tumor suppressor proteins (p19, p27, p53, and p73) were decreased in the resistant cells along with significant integrin α2, α3, and β1 alteration [201]. Such resistance mechanisms may be overcome through a strategy that uses combination therapies to sensitize cells to mTOR inhibitors. Indeed, this principle has been demonstrated in breast cancer in a recent study that used an EGFR/HER2/VEGFR multi-RTK inhibitor (AEE788) in combination with mTOR rapalogs (rapamycin, temsirolimus, or everolimus) to re-sensitize triple-negative breast cancer cells to mTOR-targeted inhibition [202]. Another study, also in breast cancer, suggested that cells resistant to everolimus could be re-sensitized by downregulating survivin [203]. 

A second future approach is the use of leveraging metabolic cellular derangements to target cancer cells. One opportunity may be related to loss of argininosuccinate synthetase (ASS1), a rate-limiting enzyme in the synthesis of arginine, in bladder cancer [204]. Loss of ASS1 has been found in many cancers that are addicted to extracellular arginine for survival, including a large proportion of bladder cancers [205]. Additional work has shown that ASS1 deficiency reduced the expression of DEPTOR, an endogenous inhibitor associated with both mTORC1 and mTORC2, through epigenetic regulation [206]. Targeting of the arginine pathway may be undertaken through use of ADI-PEG 20, which can chelate extracellular arginine and is being used in clinical trials for other cancers, and possibly in combination with mTOR-targeted approaches [207]. 

As we continue to learn more about mTOR-related signaling in UC, additional synergistic approaches may be an option for treatment of this disease. 

## 10. Conclusions

The mTOR pathway is undoubtedly extremely important for cell proliferation, survival, and metabolism, and can play a critical role in tumor initiation and progression, therapy resistance, and poor outcomes. Various components of this pathway have been shown to be altered in UC and lead to poorer prognosis. While challenges have been encountered so far regarding mTOR inhibition in UC, there have been promising studies to suggest that combination therapies could potentially be more promising than single agents, if tolerated. Continuing studies to better understand the mTOR pathway, and upstream and downstream signals, can increase our knowledge and enable further development of safe and effective treatments for this very challenging disease.
